# Evaluation of non-thyroidal illness syndrome in shock patients admitted to pediatric intensive care unit in a developing country

**DOI:** 10.1007/s00431-023-05338-w

**Published:** 2023-11-23

**Authors:** Ahmed El-Nawawy, Reham Abdel Haleem Abo Elwafa, Ahmed Khalil Abouahmed, Rehab Atef Rasheed, Omneya Magdy Omar

**Affiliations:** 1 Department of Pediatrics, Faculty of Medicine, Alexandria University, Alexandria, 21321 Egypt; 2 Department of Clinical and Chemical Pathology, Faculty of Medicine, Alexandria University, Alexandria, 21321 Egypt

**Keywords:** Non-thyroidal illness syndrome, Shock, Pediatric intensive care unit, PIM2

## Abstract

During critical illness, children my experience various changes in their thyroid hormone levels. Such changes are termed non-thyroidal illness syndrome (NTI). The extent of change correlates with the severity of the illness and its outcomes in critically ill patients. This study aimed to investigate the correlation between the severity of shock and thyroid hormone derangement. This prospective observational study included forty patients aged one month to five years who were admitted to the pediatric intensive care unit (PICU) with shock. Thyroid function tests were conducted on admission, after shock reversal, and five days later. NTI patterns were observed in 70% of patients. The PIM2 score showed a significant negative correlation with T3 (*r* =  − 0.353, *p* = 0.026) and FT3 levels on admission (*r* =  − 0.417, *p* = 0.007). Furthermore, after shock reversal, the PIM2 score continued to exhibit significant negative correlations with T4 (*r* =  − 0.444, *p* = 0.004), T3 (*r* =  − 0.329, *p* = 0.038), FT3 (*r* =  − 0.355, *p* = 0.025), and FT4 levels (*r* =  − 0.379, *p* = 0.016).

*Conclusion*: This study underscores the high prevalence of NTI in PICU shock patients and suggests monitoring thyroid hormone levels for outcome prediction and treatment guidance. Further research is needed to optimize NTI management in critically ill children.

**Table:** 

**What is Known:** *• Non-thyroidal illness syndrome (NTIS) is a condition observed in critically ill patients.* *• There has been limited research on NTI in children, and existing studies have generated conflicting results regarding the relationship between thyroid hormones and clinical outcomes in cases of sepsis and septic shock.*	
**What is New:** *• The study has revealed dynamic changes in free triiodothyronine (FT3) levels during the process of shock reversal and recovery in children who experienced shock.* *• A significant negative correlation was found between the Pediatric Index of Mortality 2 (PIM2) score and several thyroid hormone levels, including FT3 on admission and T4, FT3, and FT4 on shock reversal.*

**Supplementary Information:**

The online version contains supplementary material available at 10.1007/s00431-023-05338-w.

## Introduction

In the context of acute illness, numerous changes in thyroid hormones occur. The earliest change in the hypothalamic-pituitary-thyroid axis is a reduction in triiodothyronine (T3) production resulting from inhibition of the deiodination of thyroxine (T4) to T3, with a reciprocate increment in reverse T3 (rT3). However, in cases of prolonged critical illness, pulsatile thyroid-stimulating hormone (TSH) secretion becomes suppressed, leading to a decrease in serum T4 levels [1]. These collective changes, often referred to as “low T3 syndrome,” “non-thyroidal illness syndrome” (NTI), or “sick euthyroid syndrome” have been reported in various conditions such as infection, burns, malignancy, and myocardial infarction [2].

It has been suggested that thyroid function derangements in severe illness follow a biphasic course [3]. Initially, there is a decline in T3 levels, which appears to be an adaptive mechanism aimed at reducing catabolism and energy expenditure. However, this adaptive phase is followed by a maladaptive phase, which arises due to the unique circumstances surrounding critical illness and the resuscitative efforts applied during an extended stay in the intensive care unit. During this maladaptive phase, there is a suppression of thyroid and other pituitary hormones, accompanied by various other physiological changes [4].

The underlying causes of NTI are multifactorial and can vary depending on the specific illness. Evidently, renal and liver disease changes are specifically different from those in other types of illness. Stress also induces glucocorticoid surge, suppressing TSH and serum T4 and T3 hormone levels [5, 6]. Therefore, this stress-induced surge may contribute to the modulation of thyrotropin-releasing hormone and TSH production [7].

Pediatric shock is a common emergency that contributes to high morbidity and mortality rates [8, 9]. It is estimated that there are approximately 400,000–500,000 reported cases of pediatric septic shock worldwide annually [10].

There is a limited number of pediatric studies on NTI in children, and they often present conflicting findings regarding the relationship between thyroid hormones and the clinical outcomes of sepsis and septic shock [11–13]. While the use of thyroid hormone treatment in patients with NTIS is extensively debated in adult studies, the same discussions continue regarding its application in pediatric cases [14]. Despite the progress in diagnosing and managing pediatric shock, mortality is still high, and the involvement of NTI should be investigated.

The study aimed to investigate the correlation between the level of thyroid hormone derangement and the severity of shock, as evaluated by the pediatric index of mortality 2 (PIM2) [15] and pediatric logistic organ dysfunction 2 (PELOD2) scores [16]. Additionally, the study aimed to assess the impact of thyroid hormone levels on the length of pediatric intensive care unit (PICU) stay, the duration of shock reversal, and the changes in hormone levels after shock reversal.

## Materials and methods

This prospective observational study included 40 patients aged between one month and five years who were admitted to PICU with a new episode of shock either from the emergency department or proceeding from clinical wards from 1 January 2019 to 31 December 2019. The study was conducted at Alexandria University PICU which is located in a tertiary care teaching hospital (El-Shatby Children’s Hospital) belonging to Alexandria University Hospitals. The pediatric hospital has a total of 220 beds, including a nine-bedded intensive care unit. The PICU admits an average of 300–350 cases annually. Ethical approval for the study was obtained from the local Faculty of Medicine Ethics Committee (IRB code 00012098‑FWA: No. 00018699. Ethics approval number: No. 0105784). Written informed consent was obtained from the patients’ parents or legal guardians concerning all the procedures applied in PICU.

The shock was defined according to Pediatric Advanced Life Support, 2015 American Heart Association guidelines update for cardiopulmonary resuscitation [17].

The minimal sample size was calculated based on a previous study to investigate the time course of thyroid function, factors affecting it, and its relationship to outcomes in children surviving meningococcal septic shock. den Brinker et al. [13] concluded that children surviving meningococcal septic shock showed signs of euthyroid sick syndrome on admission. Thyroid hormone level changes in the first 24 h were prognostic for the length of the PICU stay. Based on the finding, fifty out of 304 (16.44%) patients admitted to Alexandria PICU during 2018 presented with shock. A minimum sample size of 40 critically ill children admitted to PICU with shock for this prospective cohort study with a power of 90% “power of 90% (β = 0.10),” precision of 5%, and level of significance = 95% (α = 0.05) [18, 19]. Sample size do not need to be increased to control for attrition bias [20].

Exclusion criteria comprised children with a family history of thyroid disease, underlying hypothalamic or pituitary disorders, brain injury, surgery, tumor, liver or kidney disease before PICU admission, recent chemotherapy, radiotherapy, or use of thyrostatic agents in the past six months (e.g., amiodarone, lithium, glucocorticoids, salicylates, frusemide, ferrous sulfate, phenytoin, carbamazepine, or phenobarbital). Additionally, children who died before obtaining the second or third sample or those discharged within 48 hours of admission were excluded.

All patients’ characteristics were recorded including age, sex, weight, height, and date of admission.

All admission and follow-up date were recorded through history taking, clinical evaluation on admission using PIM2 score [15], and clinical follow up by using PELOD 2 score [16].

Details of received medication such as glucocorticoids, dopamine, amiodarone, furosemide, heparin, phenytoin, carbamazepine, fenclofenac, and salicylates were recorded.

Routine cultures and laboratory investigations are conducted upon admission and repeated as necessary. These investigations include a complete blood count, electrolyte levels, C-reactive protein, serum lactate, random blood glucose, and arterial blood gases. Liver function tests, including serum aspartate aminotransferase (AST) and serum alanine aminotransferase (ALT), along with renal function tests, including serum creatinine (sCr) and blood urea nitrogen (BUN), are also performed. The Kidney Disease Improving Global Outcomes (KDIGO) definition is used to identify patients with acute kidney injury [21].

An initial sample (A) was collected from all children upon admission. This was followed by two subsequent follow-up samples: one (B) taken immediately upon shock reversal [22] defined as the maintenance of systolic blood pressure (SBP) above the 5th centile for age or above 70 mmHg for children aged 1 month to 1 year [(age × 2) + 70] for those aged 1 to 10 years, and an SBP of at least 90 mmHg for children older than 10 years. Shock reversal was considered achieved when there was no need for vasopressor support for at least 24 hours. The third sample (C) was obtained five days after shock reversal. All samples were promptly centrifuged, and the sera were separated and frozen at -8°C.

The initial (A) and last samples (C) were tested for TSH, T3, T4, free triiodothyronine (FT3), free thyroxine (FT4), and rT3 and the middle sample (B) was tested for TSH, T3, T4, FT3, and FT4.

Thyroid function tests (T3, T4, FT3, FT4, TSH) were analyzed on ADVIA Centaur XP Immunoassay system [23] (Siemens Healthcare GmbH, Germany) using direct chemiluminescence technology, while reverse T3 was analyzed on Stat Fax Microplate washer that uses enzyme-linked immunosorbent assays (ELISA) technique (Cloud Clone Corp. Houston TX USA); we assessed intra and inter assay coefficients of variation (CV), within-run% CV for TSH ranged from 2.1% to 4.9%, FT4 from 1.68% to 2.22%, FT3 from 1.7% to 4%, T4 from 1.5% to 4%, T3 from 1.5% to 2.6%, and rT3 from 2.4% to 8.1%. Similarly, run-to-run %CV for these hormones varied with TSH ranging from 1.5% to 4.4%, FT4 from 1.42% to 3.48%, FT3 from 3.1% to 3.4%, T4 from 2.7% to 5%, T3 from 3% to 6.2%, and rT3 showing a total %CV ranging from 7% to 11.5%. These coefficients of variation offer valuable insights into the accuracy and consistency of our hormone measurements, bolstering the robustness of our analytical methods [24].

### Study protocol

All patients referred from the emergency department or the clinical wards were evaluated by PICU rapid response team. The ABCs of resuscitation (airway, breathing and circulation) were evaluated and stabilized for all patients [25].

The therapeutic endpoints for resuscitation of shock include achieving a capillary refill time of ≤ 2 s, maintaining normal blood pressure for the patient’s age, having normal pulses without a difference in in pulse quality between peripheral and central, warm extremities, a urine output of > 1 ml/kg/hr, improved mental status, appropriate levels of glucose and ionized calcium concentration, a perfusion pressure that is suitable for the patient’s age, and superior vena cava O2 saturation ≥ 70% [26].

### Statistical analysis

Statistical analysis was conducted using SPSS program version 22 (Statistical Package of social sciences, Released 2013. IBM SPSS Statistics for Windows, Version 22.0. Armonk, NY: IBM Corp). Quantitative variables were summarized as mean and standard deviation and standard error of the mean. The repeated measures ANOVA were used to detect any overall differences between the three related means. Greenhouse-Geisser correction was used whenever the assumption of sphericity had been violated (tested by Mauchly’s Test of Sphericity); when there was an overall significant difference, pair-wise comparison (post hoc test) was done using a paired sample *t*-test with Bonferroni correction. Effect size for repeated measures ANOVA was estimated by Partial eta squared. The Pearson’s coefficient was used to measure the strength of correlations between two normally distributed quantitative variables. Nominal variables were summarized in the form of frequency and percentages. The significance of the obtained results was judged at the 5% level of significance [27].

## Results

**Fig. 1:**
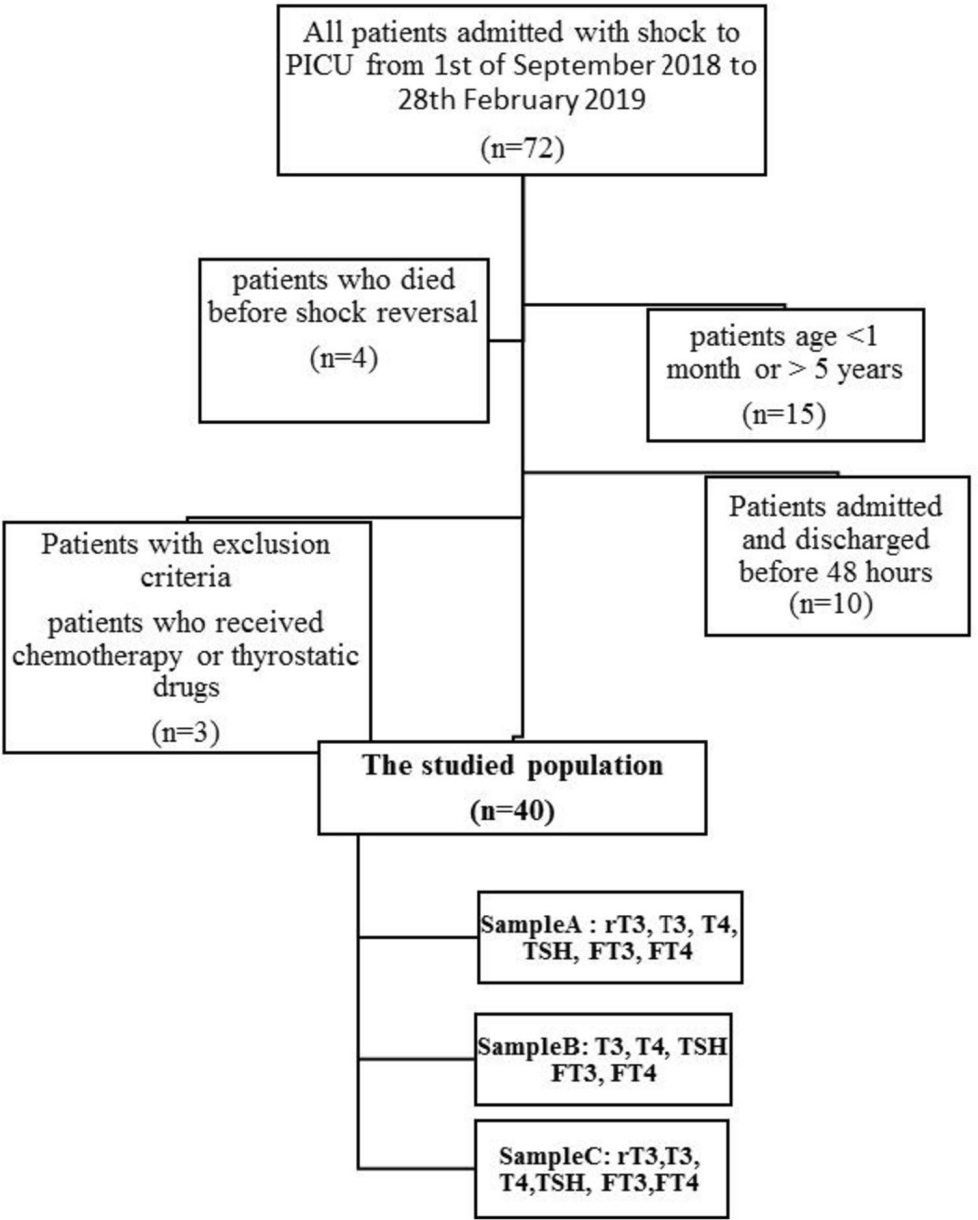
RECORD flow diagram of recruitment of the studied population

The RECORD flow diagram for the study is depicted in Fig. 1. This prospective study included patients admitted to the PICU with different diagnostic categories. Seventy-two patients with new onset shock were admitted to the PICU, and 32 were excluded. Therefore, the final sample included 40 patients. Table 1 shows their basic demographic, clinical characteristics, and the medication received.

**Table 1:** Distribution of the studied cases according to demographic, clinical observation data, and the medication received (*n* = 40)

**Sex**	
Male	21 (52.5**%**
Female	19 (47.5%)
**Age (months)**	
Min.–max.	1.20–58.0
Mean ± SD.	15.48 ± 18.43
SED	2.91
**Weight(kg)**	
Min.–max.	3.5–20.0
Mean ± SD.	8.6 ± 4.86
SED	0.77
**Weight (*Z* score)**	
Min.–max.	− 4.08–1.1
Mean ± SD.	− 1.19 ± 1.45
SED	0.23
**Height(cm)**	
Min.–max.	49.00–112.00
Mean ± SD.	71.20 ± 17.90
SED	2.83
**Height (*Z* score)**	
Min.–max.	− 5.18–2.22
Mean ± SD.	− 1.16 ± 1.59
SED	0.25
**PIM2**	
Min.–max.	7.00–95.00
Mean ± SD.	33.86 ± 23.16
SED	3.66
**MV (days)**	
Min.–max.	0.00–18.00
Mean ± SD.	5.35 ± 4.24
SED	0.67
**LOS (days)**	
Min.–max.	4.00–45.00
Mean ± SD.	15.48 ± 10.90
SED	1.72
**PELOD2 A**	
Min.–max.	2.00–15.00
Mean ± SD.	7.55 ± 2.79
SED	0.44
**PELOD2 B**	
Min.–max.	0.00–11.00
Mean ± SD.	4.80 ± 2.29
SED	0.36
**PELOD2 C**	
Min.–max.	0.00–7.00
Mean ± SD.	2.40 ± 1.85
SED	0.29
**Reversal of shock (in days)**	
Min.–max.	1.00–20.00
Mean ± SD.	5.23 ± 4.05
SED	0.64
**Medications received during their PICU stay**	
**Glucocorticoids**	22 (55%)
**Furosemide**	10 (25%)
**Phenobarbitone**	4 (10%)
**Phenytoin**	9 (22.5%)
**Heparin**	5 (12.5%)
**Inotrope**	
**Single inotrope**	22 (55%)
**Two inotropes**	11 (27.5%)
**Three inotropes**	7 (17.5%)

*LOS* length of stay, *MV* mechanical ventilation, *PELOD* pediatric logistic organ dysfunction, *PIM2* pediatric index of mortality 2, *A* on admission, *B* on shock reversal, *C* five days after shock reversal, *SED* standard error of the mean

Low FT3 levels were found in 33 patients (82.5%), while the remaining seven patients (17.5%) had FT3 levels within the normal reference range. Among the patients, 23 (57.5%) had FT4 levels within the normal reference range, while 15 patients (37.5%) had FT4 levels below the lower reference range. TSH levels within the normal reference range were observed in 26 patients (65%), with 13 patients (32.5%) having TSH levels below the lower reference range. All patients (100%) had rT3 levels above the normal reference range of 6.9–26.2 ng/dl. On shock reversal, 77.5% of patients had FT3 levels below the reference range, 12.5% had FT4 levels below the reference range, and 25% had TSH levels below the reference range. Additionally, 40 patients (100%) still had rT3 levels higher than the normal range. Five days after shock reversal, FT3, FT4, and TSH levels below the reference range were found at 72.5%, 10% and 27.5%, respectively (Table 2).

**Table 2:** Distribution of the study population hormonal status according to the reference range for age and sex of each individual

	**Low *n* (%)**	**Normal *n* (%)**	**High *n* (%)**
**T3 A**	17 (42.50%)	23 (57.50%)	0.00 (0.00%)
**T3 B**	14 (35.00%)	26 (65.00%)	0.00 (0.00%)
**T3 C**	12 (30.00%)	28 (70.00%)	0.00 (0.00%)
**T4 A**	27 (67.50%)	12 (30.00%)	1 (2.50%)
**T4 B**	19 (47.50%)	20 (50.00%)	1 (2.50%)
**T4 C**	16 (40.00%)	23 (57.50%)	1 (2.50%)
**FT3 A**	33 (82.50%)	7 (17.50%)	0 (0%)
**FT3 B**	31 (77.50%)	9 (22.50%)	0 (0%)
**FT3 C**	29 (72.50%)	11 (27.50%)	0 (0%)
**FT4 A**	15 (37.50%)	23 (57.50%)	2 (5.00%)
**FT4 B**	5 (12.50%)	32 (80.00%)	3 (7.50%)
**FT4 C**	4 (10.00%)	32 (80.00%)	4 (10.00%)
**TSH A**	13 (32.50%)	26 (65.00%)	1 (2.50%)
**TSH B**	10 (25.00%)	25 (62.50%)	5 (12.50%)
**TSH C**	11 (27.50%)	25 (62.50%)	4 (10.00%)
**rT3 A**	0 (0%)	0 (0%)	40 (100%)
**rT3 C**	0 (0%)	0 (0%)	40 (100%)

*FT3* free triiodothyronine, *FT4* free thyroxine, *rT3* reverse triiodothyronine, *T3* triiodothyronine, *T4* thyroxine, *TSH* thyroid stimulating hormone, *A* on admission, *B* on shock reversal, *C* five days after shock reversal

Non-thyroidal illness syndrome was identified in 28 patients (70%) in this study. Some patients had abnormal levels in only one hormone, while others had combinations of hormone abnormalities. The most prevalent NTI pattern observed was characterized by low levels of both FT3 and FT4, along with normal TSH levels. This pattern was found in 9 patients (22.50%). On shock reversal, the most frequent NTI pattern observed was characterized by low FT3, normal FT4, and low TSH levels, observed in 9 patients (22.50%). Five days after shock reversal, the most frequent NTI pattern observed was isolated low FT3, found in 7 patients (17.50%) (Table 3).

**Table 3:** Comparison between the non-thyroidal illness syndrome (NTI) patterns of the samples A, B, and C

**NTI patterns (FT3, FT4, and TSH)**	**A**	**B**	**C**
**Low FT3, normal FT4, and normal TSH**	3 (7.50%)	0 (0.00%)	7 (17.50%)
**Low FT3, low FT4, and normal TSH**	9 (22.50%)	2 (5.00%)	1 (2.50%)
**Low FT3, low FT4, and low TSH**	2 (5.00%)	2 (5.00%)	2 (5.00%)
**Low FT3, normal FT4, and low TSH**	3 (7.50%)	9 (22.50%)	1 (2.50%)
**Low FT3, normal FT4, and high TSH**	0 (0.00%)	1 (2.50%)	1 (2.50%)
**Normal FT3, normal FT4, and low TSH**	6 (15.00%)	5 (12.50%)	7 (17.50%)
**Normal FT3, low FT4, and low TSH**	1 (2.50%)	0 (0.00%)	0 (0.00%)
**Normal FT3, low FT4 and normal TSH**	3 (7.50%)	1 (2.50%)	0 (0.00%)
**Normal FT3, high FT4 and low TSH**	1 (2.50%)	0 (0.00%)	1 (2.50%)

*FT3* free triiodothyronine, *FT4* free thyroxine, *TSH* thyroid stimulating hormone, *A* on admission, *B* on shock reversal, *C* five days after shock reversal

By comparing the means of samples A, B, and C, it was observed that the mean of T4 in sample B was significantly higher than in sample A (*p*_1_ = 0.017), and the mean in sample C was significantly higher than sample A (*p*_2_ = 0.028). Additionally, the means for FT3 and FT4 in sample C were significantly higher than in sample A (*p*_2_ = 0.025) and (*p*_2_ = 0.005), respectively. Furthermore, the mean of TSH in sample C was significantly higher than in sample B (*p*_1_ = 0.017) and in sample C was significantly higher than in sample A (*p*_2_ = 0.015) (Table S1). However, there was no significant difference in the mean of rT3 between samples A and C (*p* = 0.132).

The dynamic nature of FT3 levels during the course of shock reversal and recovery, with patients transitioning between different FT3 level categories, is presented in Table 4.

**Table 4:** Dynamic nature of FT3 levels during the course of shock reversal and recovery

**FT3 change level**	**On admission and on shock reversal**	**On shock reversal and 5 days after shock reversal**
**Remain normal**	21 (52.50%)	25 (62.50%)
**Normal to low**	2 (5.00%)	1 (2.50%)
**Normal to high**	0 (0.00%)	0 (0.00%)
**Remain low**	12 (30.00%)	11 (27.50%)
**Low to normal**	5 (12.50%)	3 (7.50%)
**Low to high**	0 (0.00%)	5 (12.50%)

The PIM2 score had a significant negative correlation with T3 and FT3 levels on admission and with T4, FT3, and FT4 levels after shock reversal. BUN levels were significantly negatively correlated with T3 and FT3 levels after shock reversal, as well as with T3, T4, and FT3 levels five days after shock reversal. No significant correlations were found between the thyroid hormones and the medications administered to the study population during their PICU stay, CRP levels, leucocyte count, length of stay, and the duration until shock reversal (Table 5). We found no statistically significant correlations between lactate values and any of the thyroid hormones measured.

**Table 5:** Correlation between the different parameters

**Variables**	**Unadjusted correlation** **[df = 40]**		**Correlation adjusted for age** **[df (adj for age) = 37]**	
	***r***	***p***	***r* (adj)**	***p* (adj)**
**T3 A vs. PIM2**	− 0.353	0.026*	− 0.29	0.073
**T3 B vs. PIM2**	− 0.329	0.038*	− 0.272	0.094
**T3 B vs. BUN B**	− 0.468	0.002*	− 0.364	0.023*
**T3 C vs. BUN C**	− 0.377	0.017*	− 0.404	0.011*
**T4 A vs. sCr A**	− 0.320	0.044*	− 0.236	0.148
**T4 B vs. PIM2**	− 0.444	0.004*	− 0.395	0.013*
**T4 C vs. sCr C**	− 0.329	0.038*	− 0.273	0.093
**T4 C vs. BUN C**	− 0.370	0.019*	− 0.325	0.043*
**FT3 A vs. PIM2**	− 0.417	0.007*	− 0.359	0.025*
**FT3 B vs. PIM2**	− 0.355	0.025*	− 0.324	0.044*
**FT3 B vs. BUN B**	− 0.458	0.003*	− 0.356	0.026*
**FT3 C vs. BUN C**	− 0.328	0.039*	− 0.364	0.023*
**FT4 B vs. PIM2**	− 0.379	0.016*	− 0.324	0.044*
**TSH B vs. PIM2**	− 0.335	0.035*	− 0.295	0.068
**MV days vs. PIM2**	0.329	0.038*	0.373	0.019*
**MV days vs. PICU days**	0.657	< 0.001*	0.651	< 0.001*
**MV days vs. PELOD2 A**	0.447	0.004*	0.438	0.005*
**PICU days vs. lactate A**	0.378	0.016*	0.344	0.032*
**PICU days vs. lactate C**	0.353	0.026*	0.315	0.051
**PICU days vs. PELOD2 A**	0.578	< 0.001*	0.569	< 0.001*
**PICU days vs. PELOD2 B**	0.416	0.008*	0.445	0.005*
**PICU days vs. PELOD2 C**	0.382	0.015*	0.419	0.008*

*BUN* blood urea nitrogen, *FT3* free triiodothyronine, *FT4* free thyroxine, *LOS* length of stay, *MV* mechanical ventilation, *PELOD* pediatric logistic organ dysfunction, *PICU* pediatric intensive care unit, *T3* triiodothyronine, *T4* thyroxine, *TSH* thyroid stimulating hormone**,** *sCr* serum creatinine, *vs* versus, *df* degrees of freedom, *adj* adjusted for age, *r* Pearson’s correlation coefficient, *A* on admission, *B* on shock reversal, *C* 5 days after shock reversal

*Statistically significant at *p* < 0.05

## Discussion

In the present study, out of the total sample, 28 patients (70%) had NTI patterns. The observed patterns included various combinations of low FT3, low FT4, and low TSH levels, with percentages ranging from 2.50% to 22.50%. Specifically, 7.50% of patients had low FT3, normal FT4, and normal TSH levels, while 22.50% had low FT3, low FT4, and normal TSH levels. In 5.00% of patients, all three levels (FT3, FT4, and TSH) were low. Other observed patterns included low FT3 with normal FT4 and low TSH (7.50%), normal FT3 with low FT4 and low TSH (2.50%), and normal FT3 with low FT4 and normal TSH (7.50%). Additionally, 15% of patients had normal FT3, normal FT4, and low TSH levels.

It is noteworthy that all participants in the study had increased levels of rT3, which indicates its potential as a sensitive and early indicator for detecting acute alterations in thyroid hormone metabolism. The short half-life of rT3, approximately 3 h, further enhances its effectiveness in promptly identifying these changes [28].

In the study by El-Ella et al. [12], NTI patterns were observed in 62.9% of critically ill children. These patterns included low FT3 only (26.9%); low FT3 and FT4 (15%); low FT3, FT4, and low TSH (1.9%); low FT3 and TSH (5.8%); low FT3 with high TSH (1.9%); low TSH only (1.9%); and low FT4 only (1.9%).

However, Den Brinker et al. [13] described NTI patterns in 69 children with meningococcal sepsis, including low serum T4 levels (80%), normal FT4 levels (89%), low T3 levels (100%), high rT3 levels (89%), and low T3/rT3 ratios (100%). None of the patients had elevated TSH levels. The divergence may be explained by differences in the characteristics of the study populations.

Consistent with our findings, Zargar et al. [29] analyzed circulating T3, T4, and TSH levels in 382 adult patients with NTI. Different patterns were observed at the onset of illness, as well as in the 3rd and 24th weeks. These patterns included low T3 (29.6%); low T3 and T4 (13.1%); low T3, T4, and TSH (2.6%); high T4 (7.3%); low TSH (6.8%); high TSH (6.5%); and low T4 (1%).

Overall, the comparison highlights the variations in NTI patterns across different patient populations and age groups. These differences may be attributed to the underlying conditions, severity of illness, and individual physiological responses. Therefore, it is important to consider these variations when interpreting and applying the findings to clinical practice.

These findings provide valuable insights into the prevalence and distribution of NTI patterns in both children and adults, highlighting the complexity of thyroid hormone alterations during non-thyroidal illness. This wide variability could be attributed to various factors. These factors include differences in the age of the patients being studied, the underlying critical illness, the size of the study sample, the technique used for measuring free T3 levels, as well as other factors such as ethnicity.

In the present study, an unexpected finding was the elevated FT4 levels despite low FT3 and low or normal TSH levels. This can be explained by the fact that even small amounts of heparin administration could lead to in vitro generation of free fatty acids during extended serum dialysis, resulting in falsely increased apparent free hormone levels [30]. This is a significant issue as heparin is commonly used for thrombosis prevention in intensive care units, suggesting that this problem may be widespread and severe. Another possible explanation is that the decrease in total T3 levels is more significant than that of total T4 levels. Additionally, factors that impact thyroid hormone binding are more likely to affect T4 assays, as T4 is tightly bound to thyroxine-binding globulin compared to T3 [31].

Zargar et al. [29] concluded that despite clinical improvement in the majority of patients with NTI patterns by the third week, there was a continued decline in T3 and T4 levels. It was only after six months that significant evidence of recovery could be observed. Even at this point, although mean T4 levels were comparable to control values, T3 levels remained lower compared to controls.

In the present study, the median length of stay (LOS) was found to be 10.5 days. After the reversal of shock, the follow-up period varied for each patient and was not a fixed point in time. A third sample was taken after five days. The authors did not anticipate a complete recovery of thyroid hormone levels, but rather looked for a reassuring trend indicating a pattern of recovery.

By comparing the mean of thyroid hormones between the three samples, A, B and C, we concluded that mean T4 levels in sample B were significantly higher than sample A, and that of sample C was significantly higher than sample A. Furthermore, mean FT3 and FT4 levels were significantly higher in sample C than in sample A. Also, the mean of TSH in sample C was significantly higher than in sample B and sample A.

In our study, we observed a dynamic pattern of FT3 levels during the course of shock reversal and recovery in pediatric patients. These findings shed light on the intricate relationship between thyroid function and critical illness and provide valuable insights for clinicians and researchers.

The present study reported an increase in the percentage of patients maintaining normal FT3 levels after five days, highlighting the potential for recovery of thyroid function in the post-shock phase. Additionally, we noted a subset of patients transitioning from normal to low FT3 levels on shock reversal. While this transition was relatively small, it merits attention, as it could be indicative of thyroid dysfunction associated with the critical state. Further investigation is needed to determine the clinical implications of this transition.

Furthermore, among patients initially presenting with low FT3 levels, we observed variations in FT3 levels on shock reversal and five days afterward. Notably, a proportion of these patients experienced an increase in FT3 levels to the normal range five days after shock reversal. This intriguing finding suggests that thyroid function recovery is possible in some pediatric patients following the resolution of shock.

The PIM2 score showed significant negative correlations with FT3 and FT3on admission, as well as with T4, FT3, and FT4 on shock reversal. BUN levels were significantly negatively correlated with both T3 and FT3 on shock reversal, as well as with T3, T4, and FT3 five days after shock reversal. These findings suggest that higher PIM2 scores, indicating greater severity of illness, were associated with lower thyroid hormone levels, emphasizing the potential impact of critical illness on thyroid function.

It is worth mentioning that this study did not include patients with overt renal impairment. Still, since most of our study population suffered from septic shock or sepsis, their renal functions inevitably deteriorated. Sepsis is the most common precipitant for acute kidney injury in adults and children, and the progression of kidney disease in sepsis is a poor prognostic sign [32].

All patients in the study required vasoactive medication to maintain hemodynamic stability. Dopamine was not administered during their stay in the PICU. The inotropes used were dobutamine, noradrenaline, adrenaline, and milrinone. Among the patients, 22 (55%) received a single inotrope, 11 (27.5%) received a combination of two inotropes, and seven (17.5%) received a combination of three inotropes. Additionally, 22 patients received noradrenaline alone or in combination with another vasoactive medication.

The effects of these medications on thyroid hormones were evaluated in samples A, B, and C. It was found that dopamine and glucocorticoids suppress TSH secretion, while phenytoin and phenobarbital increase the hepatic metabolism of thyroid hormones, leading to decreased serum levels. Amiodarone and beta-adrenergic blocking agents inhibit deiodinase activity, thereby reducing T3 production. On the other hand, furosemide, heparin, and non-steroidal anti-inflammatory drugs transiently increase free thyroid hormone levels by inhibiting their binding to plasma transport proteins [33].

No significant correlations were observed between the thyroid hormones and the duration of medications administered to the study population during their PICU stay, CRP levels, WBC count, LOS, or the time until shock reversal.

However, El-Ella et al. [12] found a significant negative correlation between TSH and the LOS (*r* =  − 0.35; *p* = 0.011); they also found significant negative correlation between WBCs with both FT3 (*r* =  − 0.36; *p* = 0.002) and FT4 (*r* =  − 0.34; *p* = 0.005). However, no significant correlations were observed between thyroid hormones and CRP levels.

There was no significant correlation between the patients’ NTI patterns and either the LOS or the duration of shock reversal. On the other hand, den Brinker et al. [14] reported that T4 levels were an independent predictor of an unfavorable outcome, specifically a prolonged ICU stay.

A systematic review conducted by Angelousi et al. [11] reported that higher TSH levels, lower rT3 levels, or lower T3 and T4 levels were associated with an unfavorable outcome in patients with sepsis or septic shock. This suggests that, in some patients, thyroid hypofunction during sepsis or septic shock may independently influence the outcome of these conditions. Further studies are needed to evaluate this hypothesis in appropriately designed studies.

It is important to note that thyroid hormone assessments, like many other laboratory tests, may have a turnaround time that extends beyond the acute management phase. While our study focused on the potential clinical relevance of thyroid hormone assessments in pediatric shock patients, we acknowledge that these tests may not be immediately actionable during the acute phase of management. Therefore, their utility as real-time guidance for acute interventions may be limited.

The current study has several limitations, including being a single-center study with a small sample size. Conducting a larger multi-center study would provide stronger and more generalizable results. The study was also limited by the duration of patients; stay in the PICU, and a longer follow-up period would have provided more meaningful insights. Additionally, the exclusion of deceased subjects who did not survive to the second or third sample collection limits the understanding of mortality outcomes in relation to NTI. Furthermore, the study did not measure cortisol and cytokine values, which are important factors in understanding the underlying mechanisms of NTI. The lack of antibody testing in the assessment of thyroid function further limits the interpretation of these findings, especially in distinguishing between non-thyroidal illness and underlying thyroid dysfunction. These limitations highlight the need for further research to establish evidence-based guidelines for managing pediatric NTI in the PICU and to improve our understanding and management of thyroid dysfunction in critically ill children.

### Supplementary Information

Below is the link to the electronic supplementary material.

Supplementary file1 (DOCX 22 KB)

## Data Availability

All data used are included in this article. Further data that support the findings of this study are available from the corresponding author upon reasonable request by mail.

## Abbreviations

BUN: Blood urea nitrogen
FT3: Free triiodothyronine
FT4: Free thyroxine
LOS: Length of stay
MV: Mechanical ventilation
NTI: Non-thyroidal illness syndrome
PELOD: Pediatric logistic organ dysfunction
PICU: Pediatric intensive care unit
PIM2: Pediatric Index of Mortality 2
rT3: Reverse triiodothyronine
sCr: Serum creatinine
T3: Triiodothyronine
T4: Thyroxine
TSH: Thyroid stimulating hormone
