# Supplementary material for: Evaluation of non-thyroidal illness syndrome in shock patients admitted to pediatric intensive care unit in a developing country
Source: Eur J Pediatr. 2023 Nov 23;183(2):769–78. doi: 10.1007/s00431-023-05338-w (PMC10912421; doi:10.1007/s00431-023-05338-w)
Supplement: Supplementary file 1 — Supplementary file1 (DOCX 22 KB) [file 431_2023_5338_MOESM1_ESM.docx]

**Table S1: Comparison between the** **three periods according to different parameters (n = 40)**

|  | **A** | **B** | **C** | **F** **(df)**  **p-value** | **PEta^2^**  **Power (%)** |
| --- | --- | --- | --- | --- | --- |
| **T3 (**ng/ml) |  |  |  |  |  |
| Min. – Max. | 0.29 – 1.94 | 0.31 – 1.78 | 0.25 – 1.97 | F_(2,78)_ =2.987  *p*=0.056 | PEta^2=.071  Pw. =56.5 |
| Mean ± SD. | 0.84 ± 0.45 | 0.96 ± 0.47 | 1.03 ± 0.46 |  |  |
| Median | 0.73 | 0.86 | 1.12 |  |  |
| **T4 (**ug/dl) |  |  |  |  |  |
| Min. – Max. | 2.60 – 15.40 | 3.84 – 16.50 | 4.40 – 15.90 | F_(1.696, 66.161)_^$^ =6.427  *p*=0.004^$^ | pEta^2=0.141  Pw.=89.3 |
| Mean ± SD. | 7.32 ± 3.37 | 8.75 ± 3.37 | 8.76 ± 2.89 |  |  |
| Median | 6.75 | 7.95 | 7.95 |  |  |
| **Sig. between period** | *p*_1_=.017^*^, *p*_2_=.028^*^, *p*_3_=1.000 | | |  |  |
| **FT3 (**pg/ml) |  |  |  |  |  |
| Min. – Max. | 0.68 – 3.76 | 0.66 – 4.41 | 0.81 – 4.98 | F_(1.979, 77.162)_^$^ =4.125  *p*=0.02^$^ | pEta^2=0.096  Pw. =71.4 |
| Mean ± SD. | 1.87 ± 0.89 | 2.12 ± 1.05 | 2.38 ± 1.11 |  |  |
| Median | 1.64 | 1.95 | 2.28 |  |  |
| **Sig. between period** | *p*_1_=.449, *p*_2_=.025^*^, *p*_3_=.462 | | |  |  |
| **FT4 (**ng/dl) |  |  |  |  |  |
| Min. – Max. | 0.17 – 2.19 | 0.19 – 2.46 | 0.74 – 3.76 | F_(1.954, 76.2)_^$^ =6.196  *p*=0.003^$^ | pEta^2=0.137  Pw. =88.1 |
| Mean ± SD. | 1.12 ± 0.51 | 1.26 ± 0.47 | 1.42 ± 0.56 |  |  |
| Median | 1.17 | 1.22 | 1.33 |  |  |
| **Sig. between period** | *p*_1_=.261, *p*_2_=.005^*^, *p*_3_=.221 | | |  |  |
| **TSH (uIU/ml)** |  |  |  |  |  |
| Min. – Max. | 0.18 – 6.91 | 0.19 – 23.30 | 0.15 – 16.20 | F_(1.745, 68.063)_^$^ =6.87  *p*=0.003^$^ | pEta^2=0.15  Pw.=91.3 |
| Mean ± SD. | 2.08 ± 1.74 | 3.77 ± 4.22 | 3.65 ± 3.36 |  |  |
| Median | 1.72 | 2.85 | 3.25 |  |  |
| **Sig. between period** | *p*_1_=.017^*^, *p*_2_=.015^*^, *p*_3_=1.000 | | |  |  |

FT3 Free triiodothyronine, FT4 Free thyroxine, , T3 Triiodothyronine,T4 Thyroxine, TSH Thyroid stimulating hormone.

Sig significance, F F test (ANOVA) with repeated measures, ^$^ Greenhouse-Geisser correction for sphericity assumption violation, PEta2 Partial Eta Squared (effect size), Pw Observed Power (%);

*: Statistically significant at *p<*0.05 Sig. bet. periods were done using Post Hoc Test (Bonferroni adjusted Paired t-test test)

*p*_1_: p value for comparing between A and B

*p*_2_: p value for comparing between A and C

*p*_3_: p value for comparing between B and C

A: on admission, B: on shock reversal, C: 5 days after shock reversal.
